# Acute Confusional Migraine: Unusual Great Masquerader—Case Report and Literature Review

**DOI:** 10.1155/2020/9604924

**Published:** 2020-10-25

**Authors:** Bashar Tanous, Raad Tahtouh, Sundus Sardar, Sara Mohamed, Aseel Sukik, Mhd-Baraa Habib, Jamal Sajid, Mouhand F. H. Mohamed

**Affiliations:** ^1^Internal Medicine Residency Program, Internal Medicine Department, Hamad Medical Corporation, Doha, Qatar; ^2^Internal Medicine Department, Hamad Medical Corporation, Doha, Qatar

## Abstract

*Background*. Acute confusional migraine (ACM) is a rare variant of migraine, mainly prevalent in children and adolescents. It is not currently indexed as a distinct variant of migraine likely since only a few cases were reported in the adult population. We report a case of delayed ACM diagnosis in a young man and present a concise-related literature review. *Case Presentation.* A thirty-eight-year-old man with a past medical history of migraine, not on any treatment, presented with headaches accompanied by confusion. Over a two-year period before the current presentation, he experienced two episodes of confusion, which required hospital admission for evaluation: once mislabeled as a psychiatric illness and diagnosed as a migrainous infarct in the second hospitalization. In the current presentation, he reported a similar history of headache accompanied by confusion. The examination was remarkable for disorientation; otherwise, no focal deficit was elicited. Laboratory testing, cerebrospinal fluid, and neurological imaging were all unremarkable. His symptoms improved spontaneously within less than twenty-four hours, similar to his previous presentations. After two-year history of episodic confusion and after excluding other plausible causes of confusion, guided by proposed diagnostic criteria, we diagnosed him as a case of ACM. The patient remains well at the follow-up of two months after discharge. *Discussion and Conclusion.* ACM is a rare variant of migraine and is often a challenge for clinicians to diagnose appropriately. Until recent years, the disease was thought to be limited to children and adolescents. However, recently few reports also expanded the incidence of this entity to the adult population. There is a significant gap in knowledge about proper identification and treatment of this condition, leading to delayed or overlooked ACM diagnosis. Moreover, the recent edition of the International Classification of Headache Disorders (ICHD-3) does not account for this entity, thereby further adding to physicians' lack of awareness regarding this migraine subtype. The authors emphasize that clinicians be aware of this entity and adequately utilize the existing proposed diagnostic criteria for ACM until standardized and validated tools are available. We also believe that this entity should be acknowledged in the subsequent migraine guidelines and classifications.

## 1. Introduction

Migraine is a widespread disease affecting over one billion individuals worldwide [1]. It leads to increased morbidity and adds to the global healthcare burden, with more than twenty million years of life lost in disability (YLD) in 2016 [1]. It is a primary headache disorder characterized by moderate-to-severe unilateral headaches, usually lasting up to 72 hours and is more common in females [2]. Migraine can occur either with aura (transient focal neurological symptoms preceding or accompanying the headache) or without aura [3].

There are numerous well-characterized subtypes of migraine mentioned in the third edition of the International Classification of Headache Disorders (ICHD-3), including, but not limited to, hemiplegic migraine, retinal migraine, menstrual migraine, vestibular migraine, chronic migraine, and migraine with brainstem aura. The ICHD-3 failed to index acute confusional migraine (ACM), a rare and highly disabling distinct subtype of migraine [4, 5]. It has been described in the pediatrics population for the last fifty years. Since its description, very few reports documented this subtype, especially in adults, deeming ACM an infrequent entity. We report a case of delayed diagnosis of ACM in a young adult man. Additionally, we present an updated literature review highlighting the typical presenting features, diagnosis, and management of ACM to enable clinicians to identify and treat this condition properly.

## 2. Case Presentation

We present the case of a 38-year-old previously healthy man who was admitted to our hospital three times over the last two years. The first encounter was in early 2018 when the patient presented with a severe headache associated with photophobia, nausea, and vomiting with subsequent slurred speech and disorientation to time, place, and person. During hospitalization, the patient could not follow commands; thus, a formal neuropsychiatric evaluation was limited. Laboratory tests' results were unremarkable (white blood cells, hemoglobin, electrolytes, toxicology screening, kidney and liver functions, etc.).

**Figure 1:**
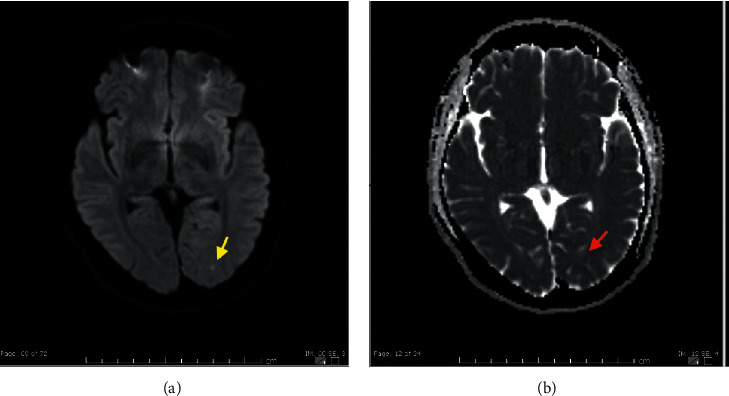
(a) Diffusion-weighted imaging (DWI) MRI head showing a tiny occipital subcortical hyperintense lesion. (b) The same lesion appearing faintly hypodense on apparent diffusion coefficient (ADC) MRI head. These findings of diffusion restriction were keeping with acute to early subacute lacunar infarction. Notably, these findings were not present on the new MRI done in the third admission (index admission).

Furthermore, a computed tomography (CT) scan of the head was normal. His confusion settled within a few hours with no specific treatment. He was then discharged from the emergency with psychiatry follow-up as a case of a functional confusional state. The patient did not attend his scheduled follow-up. One year later, he presented again with headache and confusion, resembling his earlier presentation. Examination, laboratory testing, and CT head were yet unremarkable. Magnetic resonance imaging (MRI) head revealed a left occipital tiny acute to subacute lacunar infarct, which was the only positive finding (Figure 1). The patient's symptoms resolved completely within twenty-four hours. At this time, the patient reported a fifteen-year history of migraine for which he is not on any specific therapy. Although the MRI finding did not explain the patient presentation, he was assessed to be a case of migrainous infarction (proved later to be an incorrect diagnosis) and started a short course of aspirin and statins.

In January 2020, the patient was admitted with the third episode of headache and confusion. The patient was brought to our hospital by his roommates, who stated he initially experienced severe headaches associated with photophobia, nausea, and three vomiting episodes. Soon thereafter, he developed incomprehensible slurred speech, and he became unaware of his surroundings. There was no history of head trauma, seizure, loss of sphincter control, or loss of consciousness.

On admission, his temperature was 36.8°C, heart rate, 90 beats/min, and blood pressure, 108/60 mmHg. He was confused, not following commands, restless, and disoriented. His uncooperative status limited thorough neuropsychiatric assessment. Nonetheless, there were no gross focal neurological deficits. Systemic examination was otherwise unremarkable. Laboratory blood test results were normal (Table 1). Cerebrospinal fluid (CSF) analysis was unremarkable. MRI head was normal. The remaining workup, including carotid-doppler ultrasonography, Holter monitoring, and echocardiography, was unremarkable.

The patient's hospital course was similar to his previous admissions as he returned to his baseline status within a few hours. At this stage, the information available for us was three hospitalizations (mimicking psychiatric disease and stroke) with headache and confusion settling within a few hours. All investigations were unremarkable, including MRI head. Given the exclusion of other organic causes and his stable course between his symptoms, we diagnosed him as a case of acute confusional migraine (ACM)—a rare migraine variant. The patient is well at the follow-up upon two months after discharge, occasionally taking analgesics for headache relief.

## 3. Discussion

Gascon and Barlow in 1969 described a unique confusional state that accompanied juvenile migraine [6]. Eight years later, Ehayi and Fenichel reported additional cases of five children with migraine and confusion along with their outcome, and they further coined the term “acute confusional migraine” (ACM) [7]. The prevalence of this rare condition was estimated from focused studies on pediatric migraine patients and ranged as low as 0.45% (most extensive survey of 1106 migraine patients), up to 7% (the retrospective study of 280 children and adolescents) [8, 9]. Till recently, it was thought that this rare entity is limited to children and adolescents. However, few recent reports described its occurrence in adults, suggesting expanding this entity to the adult population. The exact prevalence of this entity in the adult cohort is yet unclear. This is likely owing to the scarcity of data describing ACM in the adult population. A recent review by Farooqi et al. estimated that only 20 cases of ACM were reported in adults, making this condition extremely rare in this population [10].

The clinical presentation is variable. Personal or family history of migraine is usually present in 54% and 62% of the cases, respectively. The distinctive features are headaches, accompanied by confusion, agitation, or disorientation. These patients may also develop speech or memory disturbances, numbness, or even blindness. The neurological symptoms and the confusion usually resolve within less than 24 hours; however, the confusion may last for a few minutes up to 72 hours [10]. Laboratory workup and imaging are often unremarkable, but occasional abnormalities were previously reported. Nezu et al. reported transient cerebral hypoperfusion in the posterior cerebral artery (PCA) territory, as demonstrated by single-photon emission computerized tomography in a child with ACM [11]. Using magnetic resonance angiogram (MRA), Fujita et al. described the same phenomenon only during acute ACM attack in a child with migraine [12]. EEG demonstrated generalized slowing during ACM attacks in some cases [12], and a typical slow pattern of frontal intermittent rhythmic delta activity (FRIDA) was shown by Pietrini et al. [13]. There is no specific therapy as the condition is usually self-limiting; however, sodium valproate and topiramate have been attempted with variable efficacy for ACM treatment and prevention, respectively [10, 12].

The diagnosis can be overlooked, delayed, or missed as with our patient. Our patient presented to the emergency department on three occasions: first being misdiagnosed as a psychiatric illness; secondly, managed as migrainous infarct, another migraine variant; and lastly, as the clinical picture became apparent, the diagnosis of ACM was established (after more than two years from the initial encounter). The latest edition of the International Classification of Headache Disorders (ICHD-3) involves many rare migraine variants. Despite the knowledge of ACM for half a century, it remains unclassified. [5, 9] This, in part, contributes to the lack of awareness among front-line clinicians about this condition and its proper management. Although there are no standardized and validated criteria for ACM diagnosis, Farooqi et al. recently suggested proposed criteria that are practical and straightforward to implement in clinical practice (Table 2) [10]. We used it as a guide in establishing the diagnosis in our case. An essential pillar of these diagnostic criteria is that ACM diagnosis remains that of exclusion.

## 4. Conclusion

We presented the unusual case of confusional migraine. Our review revealed that this entity is infrequent in adults. It also showed the lack of standardized diagnostic criteria or recognizing this entity as a distinct migraine variant. Our case also highlighted the lack of proper knowledge about this entity among clinicians, which results in unnecessary overinvestigation and treatments and, thus, delays in establishing the appropriate diagnosis. MRI and EEG can be of diagnostic value during acute attacks; sodium valproate and topiramate have been used to treat and prevent this condition. Without therapy, confusion usually settles within less than 24 hours. Clinicians should be made aware of this entity, and guideline makers should consider this entity as a distinct variant of migraine.

## Figures and Tables

**Table 1:** The patient's laboratory test results.

Test (normal range/unit)	Result
WBC (4–11 x 10 ^∧^ 3/ul)	H 13.4
RBC (2.8–4.8 x 10 ^∧^ 6/ul)	5.1
Hb (12–15 gm/dL)	14.9
Platelets (150–400 x 10 ^∧^ 3/ul)	235
Urea (2.7–8 mmol/L)	3.20
Creatinine (44–80 umol/L)	68
Sodium (135–145 mmol/L)	138
Potassium (mmol/L 3.5–5.1)	3.7
Glucose (3.3–5.5 mmol/L)	8.1
ALT (05–5 U/L)	25
AST (04–0 U/L)	25
TSH (0.3–4.2 IU/L)	0.18
Free T3 (3.7–6.4 pmol/L)	5.2
Free T4 (11.6–21.9 pmol/L)	18.8
Acetaminophen (66.0-199.0)	Below level of detection
Ethanol <10,9 mmol/L	<2.2
Salicylate (150–300 mg/L)	Below level of detection
Cholesterol (<5.2 mmol/L)	4.0
Triglyceride (<1.7 mmol/L)	0.5
HDL (>1.0 mmo/L)	0.9
LDL-calc <3.36 mmol/L	2.9 mmol/L
HbA1C % 4.8-5.9	5.6 %
Lactic acid (0.5–2.2 mmol/L)	1.0
CRP (0–5 mg/L)	<5
CSF glucose (2.22–3.89 mmol/L)	3.84
CSF protein (0.15–0.45 gm/L)	0.32
CSF color	Colorless
CSF appearance	Clear
CSF WBC (0–5 uL)	1
CSF RBC^*∗*^ (0–2 uL)	460
Protein C activity (70–140%)	73.2%
Protein S activity (72–126%)	96%
Factor V	Negative factor V Leiden mutation
Anticardiolipin ab IgM/IgG	Negative
ANA	Negative

^*∗*^RBCs in the CSF were likely traumatic given the difficulty encountered while performing the lumbar puncture. WBC = white blood cells, RBC = red blood cells, Hb = hemoglobin, ALT = alanine aminotransferase, AST = aspartate aminotransferase, TSH = thyroid stimulating hormone, T3 = triiodothyronine, T4 = thyroxine, HDL = high-density lipoprotein, LDL = low-density lipoprotein, CRP = C-reactive protein, CSF = cerebrospinal fluid.

**Table 2:** Proposed criteria for the diagnosis of acute confusional migraine (By Farooqi et al. [9]).

At least one attack, fulfilling criteria A to F
A: at least one of the following: decreased attention/altered awareness/impaired cognition
B: at least one of the following: agitation or combativeness/perception disturbances/slowing or FRIDA pattern on EEG resolving within less than a week/aura lasting less than an hour
C: complete resolution in less than 24 hours or after sleep, additionally partial or complete amnesia of the event
D: normal neurological exam following the attack (no persistence of acquired neurologic deficit)
E: at least one of the following: personal history of migraine or family history of migraine or headache occurring before/during or after the confusion.
F: not attributed to another disorder (medical disorder or intoxication).

Adapted from Farooqi et al [9].

## Data Availability

Additional data used to support the findings of this study are available from the corresponding author upon request.
